# Cell-type and spatiotemporal transcriptional signatures of white matter morphometric similarity network alterations in major depressive disorder

**DOI:** 10.1017/S0033291726104711

**Published:** 2026-06-04

**Authors:** Yue Wu, Jinglei Xu, Haolin Wang, Yulong Shen, Ying Zhai, Minghuan Lei, Zhihui Zhang, Qian Wu, Qi An, Wenjie Cai, Libo Su, Yanmin Peng, Quan Zhang, Feng Liu

**Affiliations:** 1 Department of Radiology and Tianjin Key Laboratory of Functional Imaging & Tianjin Institute of Radiology, Tianjin Medical University General Hospital, Tianjin, China; 2 School of Laboratory Medicine, Division of Medical Technology, Tianjin Medical University, Tianjin, China; 3 School of Medical Technology, Tianjin Medical University, Tianjin, China; 4 School of Medical Imaging and Tianjin Key Laboratory of Functional Imaging & Tianjin Institute of Radiology, Tianjin Medical University, Tianjin, China

**Keywords:** major depressive disorder, network neuroscience, oligodendrocyte and myelination pathways, transcriptomic integration, white matter morphometric similarity networks

## Abstract

**Background:**

White matter (WM) abnormalities are implicated in major depressive disorder (MDD), yet the organization of white matter morphometric similarity networks (WM-MSNs) – which capture interregional similarity in voxel-wise WM morphology – and the transcriptional mechanisms associated with their disruption remain insufficiently understood.

**Methods:**

Using T1-weighted MRI from a large multisite sample (1,154 individuals with MDD and 1,026 healthy controls), we constructed individualized WM-MSNs. Group differences were assessed at the edge, global, and nodal levels. To identify molecular pathways underlying these alterations, nodal abnormalities were linked to regional gene expression profiles from the Allen Human Brain Atlas using spatially informed transcriptomic association, followed by functional, cell-type-specific, and developmental enrichment analyses.

**Results:**

MDD showed distributed but selective reorganization of WM-MSNs. Network-based statistics revealed two significant components, with 118 edges exhibiting increased morphometric similarity and 45 showing decreased similarity. Globally, MDD demonstrated higher small-worldness, clustering coefficient, global efficiency, and local efficiency, together with shorter characteristic path length. Nodal disruptions were concentrated in major commissural and association tracts – including the corpus callosum, cingulum, uncinate fasciculus, and tapetum. Transcriptomic integration indicated enrichment for gene signatures related to oligodendrocyte function, myelination, lipid metabolism, axonal organization, and cellular stress-related molecular processes, with implicated genes showing broad developmental-stage expression.

**Conclusions:**

MDD is associated with robust alterations in individualized WM-MSNs that converge with transcriptional signatures linked to myelination, metabolic processes, axonal structure, and cellular stress, linking macroscale network disruption to underlying molecular architecture and providing cross-scale insights into WM pathology in depression.

## Introduction

Major depressive disorder (MDD) is increasingly conceptualized as a disorder involving disturbances in large-scale brain network organization rather than isolated regional abnormalities (Marx et al., 2023; Yang et al., 2021; Zhang et al., 2025). Although extensive neuroimaging research has revealed abnormalities in both gray matter structural network organization and functional network coupling (Cai et al., 2023; Hossein et al., 2023; Li et al., 2021), white matter (WM) – the anatomical infrastructure supporting both short- and long-range communication and enabling integrative processing across distributed neural systems – has received comparatively less attention in efforts to characterize network-level pathology in MDD. Because WM pathways form the structural backbone of interregional communication, disruptions within WM architecture may contribute to the cognitive, affective, and motivational impairments that define depressive illness (Flinkenflügel et al., 2024; Huang et al., 2023). Yet despite its fundamental role in sustaining coordinated neural signaling, the macrostructural organization of WM morphology and its relationship to the pathophysiology of MDD remain insufficiently understood.

Existing work on WM in MDD has relied primarily on diffusion-based approaches, including microstructural indices from diffusion tensor imaging and tractography-based reconstructions of structural connectivity (Belge et al., 2022; Guo et al., 2012a, 2012b; Meinert et al., 2022; Repple et al., 2020). These studies have identified abnormalities within major commissural, association, and fronto-limbic pathways, such as the corpus callosum, cingulum, uncinate fasciculus, and tapetum, which are essential for emotion regulation, cognitive control, and limbic–cortical integration. However, diffusion metrics are inherently sensitive to fiber crossings, acquisition variability, and model-dependent assumptions, and they capture only a portion of the biological properties that shape WM tissue (Maier-Hein et al., 2017). Parallel research using WM structural covariance networks has examined coordinated morphometric variation across individuals (Van Etten et al., 2024; Wang, Zhao, & Li, 2022), but such group-level methods are not designed to capture the substantial individual heterogeneity characteristic of MDD. Together, these constraints underscore the need for analytic frameworks capable of characterizing WM architecture at the individual level while capturing biologically meaningful macrostructural features that extend beyond microstructural diffusion properties.

White matter morphometric similarity networks (WM-MSNs) offer such a framework. Derived from T1-weighted structural MRI, WM-MSNs quantify the voxel-wise morphometric similarity between predefined WM regions, enabling individualized and highly reproducible characterization of WM macrostructural organization (Li et al., 2024). In their study, Li and colleagues showed that morphometric similarity indices demonstrate strong test–retest reliability, stability across scanners, and substantial heritability, suggesting that WM-MSNs reflect robust and biologically grounded architectural principles. Given increasing evidence that MDD is associated with heterogeneous and individually specific alterations in WM pathways (Gai et al., 2024; Li et al., 2020), individualized WM-MSNs may reveal forms of macrostructural reorganization that remain undetected by microstructural or group-level morphometric approaches. Nevertheless, the relationship between disruptions in the network-level organization of WM morphology and the biological underpinnings of MDD remains largely unexplored. Understanding these relationships is essential for identifying how distributed WM abnormalities arise and how they contribute to the altered communication dynamics characteristic of depressive states.

**Figure 1.**
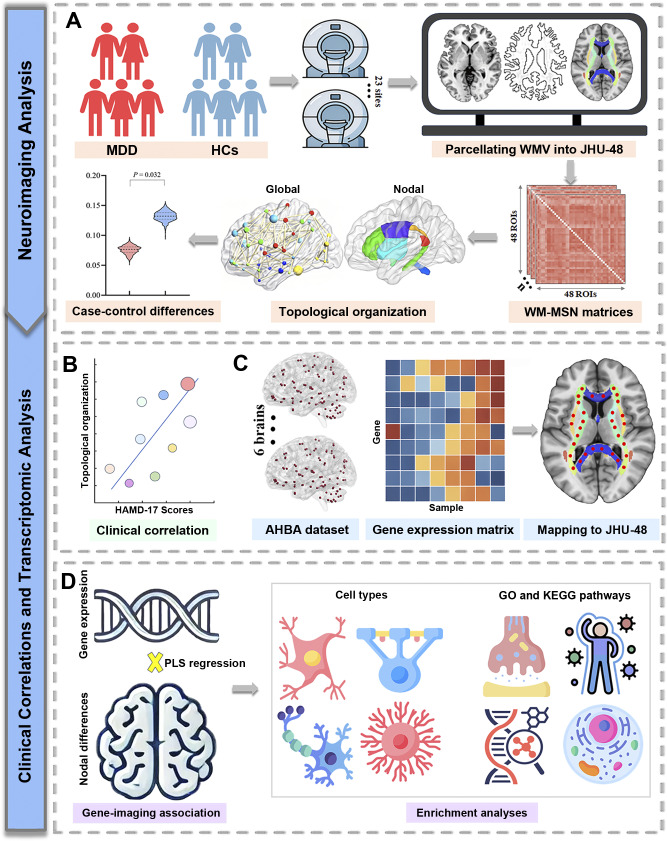
Flowchart of the study design. (a) Structural MRI data from individuals with MDD and HCs across 23 sites were used to derive WMV maps. WMV was parcellated into 48 regions according to the JHU-48 atlas, and individualized WM-MSNs were constructed based on interregional morphometric similarity. Global and nodal topological properties were then calculated, followed by case–control comparisons of network organization. (b) Associations between WM-MSN alterations and clinical severity, indexed by HAMD-17 scores, were examined. (c) AHBA transcriptomic data were processed to generate a regional gene expression matrix, and tissue samples were mapped to the JHU-48 atlas. (d) PLS regression was performed to link regional nodal alterations with gene expression profiles, followed by enrichment analyses including cell-type-specific analysis as well as GO and KEGG pathway annotation. Abbreviations: AHBA, ‘Allen Human Brain Atlas’; GO, ‘Gene Ontology’; HAMD-17, ‘17-item Hamilton Depression Rating Scale’; HCs, ‘healthy controls’; JHU, ‘Johns Hopkins University’; KEGG, ‘Kyoto Encyclopedia of Genes and Genomes’; MDD, ‘major depressive disorder’; MRI, ‘magnetic resonance imaging’; PLS, ‘partial least squares’; ROI, ‘region of interest’; WMV, ‘white matter volume’; WM-MSNs, ‘white matter morphometric similarity networks’. Figure 1. long description.

At the same time, macroscale network abnormalities are increasingly recognized to be shaped by underlying molecular and transcriptional architecture (Cai et al., 2024; Seidlitz et al., 2020; Shafiei et al., 2023). Spatial gradients of gene expression have been shown to align with cortical thinning, morphometric similarity, and functional alterations in MDD, suggesting that transcriptional organization constrains the spatial patterning of neuroimaging phenotypes across modalities (Liu, Abdellaoui, Verweij, & Van Wingen, 2023; Xue et al., 2022, 2023; Zhu et al., 2023). Whether comparable molecular mechanisms underlie WM morphometric networks – and whether WM-MSN alterations in MDD reflect specific cellular, developmental, or biological pathways – remains unknown. Addressing this gap is crucial, as mapping the transcriptional substrates of WM network disruption may illuminate how molecular processes manifest at the systems level and contribute to the complex neurobiology of depression. To this end, the present study integrates individualized WM-MSNs derived from large-scale multisite neuroimaging with spatially resolved transcriptomic data to characterize the macrostructural organization of WM in MDD and identify its underlying molecular correlates. By linking macroscale WM network organization with microscale transcriptional architecture, this work provides new cross-scale insights into the structural and molecular foundations of WM abnormalities in MDD and advances our understanding of how WM morphology contributes to depressive neuropathology. The overall study design and workflow are illustrated in Figure 1.

## Methods

### Study participants

Participants were drawn from the REST-meta-MDD Consortium (Yan et al., 2019), a large multisite collaborative initiative involving 25 imaging research centers in China. The database initially included 1,300 MDD patients and 1,128 healthy controls (HCs). Participants were excluded if they (1) lacked baseline clinical information, (2) showed poor image quality or segmentation/registration failure based on visual inspection, (3) were younger than 18 years, or (4) were recruited from sites with fewer than 10 participants in either the MDD or HC group. After quality control procedures, a total of 1,154 MDD patients and 1,026 HCs were retained for the subsequent analyses. Group differences in demographic and clinical characteristics were assessed using two-sample *t*-tests for normally distributed continuous variables and Mann–Whitney *U* tests for non-normally distributed data. Categorical variables were compared using the Pearson chi-square test.

### Construction of morphometric WM networks

Preprocessed white matter volume (WMV) maps provided by the REST-meta-MDD Consortium were used to construct individualized WM-MSNs, following previously established frameworks for morphometric brain network analysis (Li et al., 2021, 2024; Wang, Jin, Zhang, & Wang, 2016). For each participant, WMV maps were parcellated into 48 regions of interest (ROIs) according to the Johns Hopkins University (JHU) WM atlas (Supplementary Table S1). Within each ROI, voxel-wise WMV values were extracted and modeled as probability density functions using kernel density estimation. Interregional morphometric similarity was quantified using the Jensen-Shannon divergence (JSD), with lower JSD values indicating greater similarity. JSD values were then transformed into similarity measures (1 − JSD) to generate individual WM-MSN matrices ranging from 0 to 1. To remove potential site effects on interregional morphometric similarity, ComBat harmonization was applied to the individual WM-MSN similarity matrices (Cetin-Karayumak et al., 2024; Fortin et al., 2017).

### Topological analysis of morphometric WM networks

Before topological characterization, individual WM-MSNs were binarized using a sparsity-based thresholding procedure to ensure comparable network density across participants. Given the absence of a definitive criterion for selecting a single sparsity level, a consecutive sparsity range of 0.09–0.30 with an interval of 0.02 was applied. This range was selected to maintain sufficient network sparsity while preserving overall network connectedness and enabling reliable estimation of small-world topological properties (Li et al., 2024). Within this sparsity range, graph-theoretical metrics were calculated at both global and nodal levels. Global measures included normalized clustering coefficient (*γ*), normalized characteristic path length (*λ*), small-worldness (*σ*), global efficiency (Eglob), and local efficiency (Eloc), whereas nodal measures comprised degree centrality (DC), betweenness centrality (BC), and nodal efficiency (NE) for each ROI. To obtain threshold-independent summary estimates, the area under the curve (AUC) across the sparsity range was calculated for each network metric and subsequently entered into statistical analyses (Liu, Zhuo, & Yu, 2016). All network analyses were performed using the GRETNA toolbox (Wang et al., 2015).

### Group comparison and clinical association analyses of WM-MSNs

Between-group differences in WM-MSN connectivity were assessed using the network-based statistic (NBS) approach (Zalesky, Fornito, & Bullmore, 2010). For each edge, general linear models were fitted with age, sex, and years of education included as covariates. A primary component-forming threshold corresponding to *P* < 0.001 (two-tailed) was applied to the test statistics to define suprathreshold connections, and connected components were subsequently identified. The statistical significance of each component was evaluated using nonparametric permutation testing (10,000 permutations), in which group labels were randomly reassigned and the size of the largest connected component was recorded for each permutation to construct the empirical null distribution. Components were considered significant at a family-wise error (FWE)-corrected threshold of *P* < 0.05 at the network level. In addition, group differences in global small-world metrics (*γ*, *λ*, *σ*, Eglob, and Eloc) and nodal properties (DC, BC, and NE) were examined using general linear models with age, sex, and years of education included as covariates. Multiple comparisons were controlled using the false discovery rate (FDR) procedure, and results with FDR-adjusted *P* < 0.05 were considered statistically significant.

To examine the clinical relevance of network alterations, partial correlation analyses were conducted within the MDD group between Hamilton Depression Rating Scale (HAMD-17) scores and (1) the weighted connectivity strength of edges showing significant NBS effects, (2) global network metrics exhibiting significant between-group differences, and (3) nodal properties of regions demonstrating significant alterations. Age, sex, and years of education were included as covariates. Statistical significance was defined at FDR-adjusted *P* < 0.05.

### Medication-status subgroup differences

To investigate the potential effects of psychotropic medication on WM-MSN organization, subgroup analyses were performed within the first-episode MDD cohort by comparing patients receiving psychotropic medication with those not receiving medication at the time of MRI acquisition. Only individuals with complete information on medication status and illness duration were included. WM-MSNs were constructed using the same procedures as in the main analysis. Group differences were evaluated at the edge, global, and nodal levels using the same statistical framework and significance criteria as in the main case–control analysis, with illness duration additionally included as a covariate.

### Transcriptome-neuroimaging association analysis

Gene expression data were obtained from the Allen Human Brain Atlas (AHBA; Hawrylycz et al., 2012), comprising microarray-based transcriptional profiles from 3,702 tissue samples across six adult donors (Supplementary Table S2). The *abagen* toolbox was used to preprocess the data following established procedures (Markello et al., 2021). Briefly, probes were reannotated to updated gene symbols, and those with low expression intensity (below background in ≥50% of samples) were discarded. For each gene, the probe with the highest differential stability across donors was retained. Tissue samples were assigned to WM regions defined by the JHU-48 atlas, and samples located more than 2 mm from the assigned region were excluded. Gene expression values were normalized within each donor using scaled robust sigmoid normalization. Samples mapped to the same region were averaged within each donor and then across donors to generate a regional gene expression matrix (48 regions × 15,633 genes). Regions without available expression samples were excluded, resulting in 40 WM regions retained for subsequent analyses.

Partial least squares (PLS) regression was used to identify transcriptional correlates of WM network alterations by assessing the spatial correspondence between regional gene expression profiles and multivariate nodal alteration patterns (Abdi & Williams, 2013). Regional case–control differences (i.e. *t* values) in BC, DC, and NE were jointly included as response variables, while the regional gene expression matrix served as the predictor set. To evaluate statistical significance while accounting for spatial autocorrelation, spatially constrained surrogate maps were generated using BrainSMASH (1,000 iterations) (Burt et al., 2020). The proportion of variance explained in the nodal alteration patterns by each latent component was compared against the corresponding null distribution. When more than one latent component reached permutation-based significance (*P*_perm_ < 0.05), the component explaining the largest proportion of variance in the nodal alteration patterns was selected for subsequent analyses. Gene contributions were further assessed using bootstrapping (1,000 iterations), and normalized gene weights (*Z*-scores) were calculated by dividing each gene loading by its bootstrap-derived standard error (Ma et al., 2023; Xue et al., 2025). Genes surviving FDR correction (*P* < 0.05) were considered significantly associated with WM network alterations and were included in downstream analyses.

Functional enrichment analyses were performed using Gene Ontology (GO; including biological process, cellular component, and molecular function categories) and Kyoto Encyclopedia of Genes and Genomes (KEGG) pathways via Metascape (https://metascape.org). In addition, cell-type-specific expression analysis (CSEA) and spatiotemporal expression analysis (https://doughertytools.wustl.edu/CSEAtool.html) were conducted to explore potential cellular and developmental mechanisms underlying the observed transcriptional associations. Statistical significance was defined at FDR-adjusted *P* < 0.05.

### Validation analyses

To evaluate the robustness of the WM-MSN findings, two complementary validation analyses were conducted. First, to examine the potential influence of spatial smoothing on network construction, individual WMV images were smoothed using an 8-mm full-width at half-maximum Gaussian kernel, and WM-MSNs were reconstructed following the same procedures as in the primary analysis. Group-averaged WM-MSN matrices derived from smoothed and unsmoothed data were compared using spatial Pearson correlation within each group. Network measures at the edge, global, and nodal levels were subsequently re-examined using the same statistical framework as in the primary analysis. Second, to assess the stability of results with respect to model specification, all analyses were additionally repeated without covariate adjustment.

## Results

### Demographic and clinical characteristics

The final study sample comprised 1,154 patients with MDD (736 females) and 1,026 healthy controls (HCs; 603 females) recruited from 23 imaging centers after quality control procedures. Sex distribution differed significantly between groups (Pearson chi-square test, *P* = 0.017), with a higher proportion of females in the MDD group. Participant age distributions are shown in Supplementary Figure S1, and no significant between-group difference in age was observed. Regarding years of education, the MDD group had a median of 12 years (IQR = 8–15), whereas the HC group had a median of 13 years (IQR = 9–16). Patients with MDD had significantly fewer years of education than HCs (Mann–Whitney *U* test, *P* < 0.001).

### Altered WM-MSN organization in MDD

**Figure 2.**
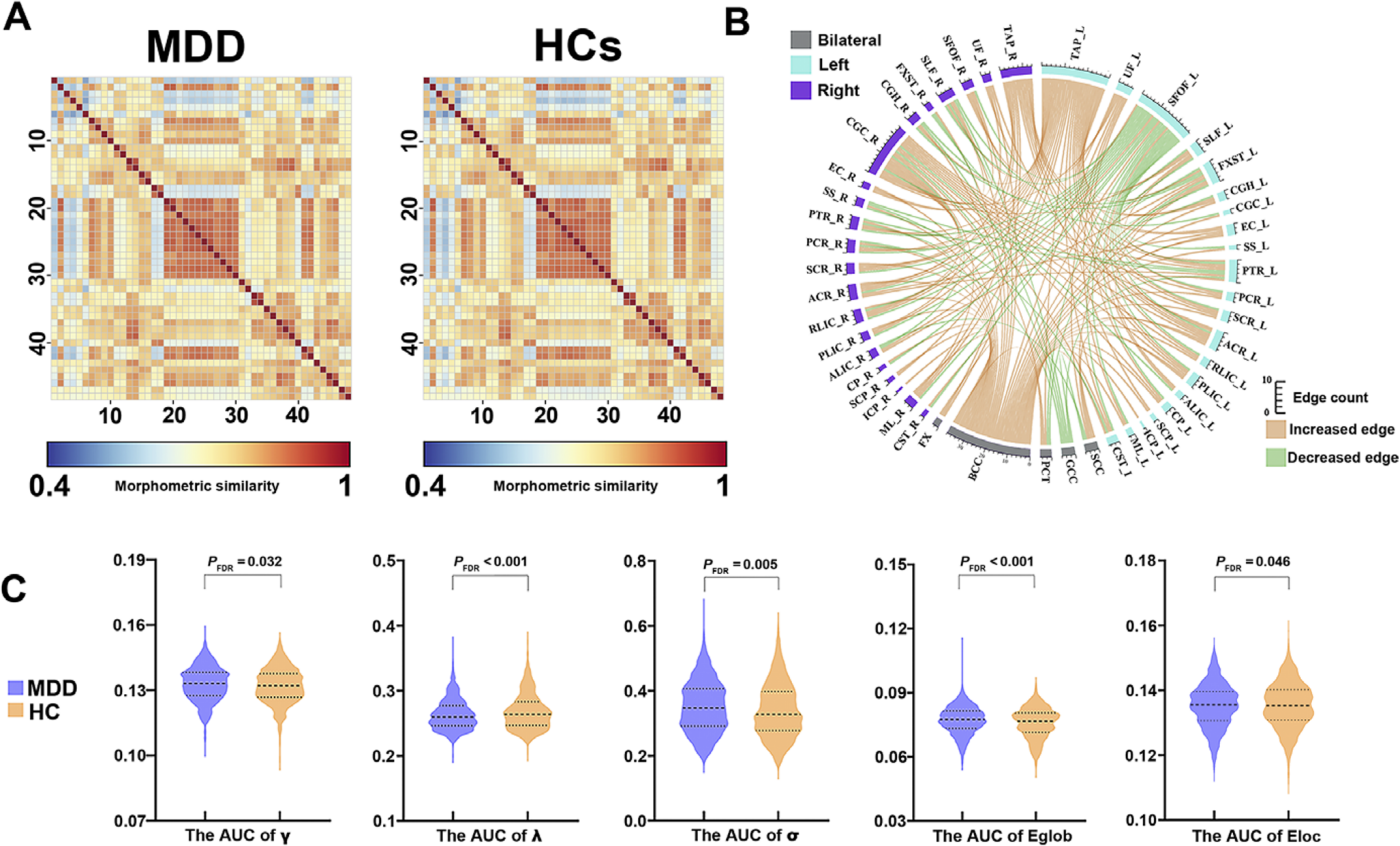
Case–control differences in WM-MSNs and global topological properties. (a) Group-averaged WM morphometric similarity matrices for the MDD and HCs groups based on the JHU WM atlas. Each matrix element represents the interregional morphometric similarity between pairs of WM regions. (b) NBS analysis identified significant between-group alterations in WM morphometric similarity. ROIs are colored according to laterality, with gray indicating bilateral regions, cyan indicating left-hemisphere regions, and purple indicating right-hemisphere regions. Orange edges indicate increased similarity in MDD relative to HCs, whereas green edges indicate decreased similarity in MDD. The length of each ROI segment reflects the number of altered edges connected to that region. (c) Violin plots showing between-group differences in global topological metrics. *P* values were derived from general linear models adjusted for age, sex, and years of education, with FDR correction for multiple comparisons. Abbreviations: AUC, ‘area under the curve’; Eglob, ‘global efficiency’; Eloc, ‘local efficiency’; HCs, ‘healthy controls’; JHU, ‘Johns Hopkins University’; MDD, ‘major depressive disorder’; NBS, ‘network-based statistic’; ROI, ‘region of interest’; WM, ‘white matter’; WM-MSNs, ‘white matter morphometric similarity networks’; *γ*, ‘normalized clustering coefficient’; *λ*, ‘normalized characteristic path length’; *σ*, ‘small-worldness’. Figure 2. long description.

**Figure 3.**
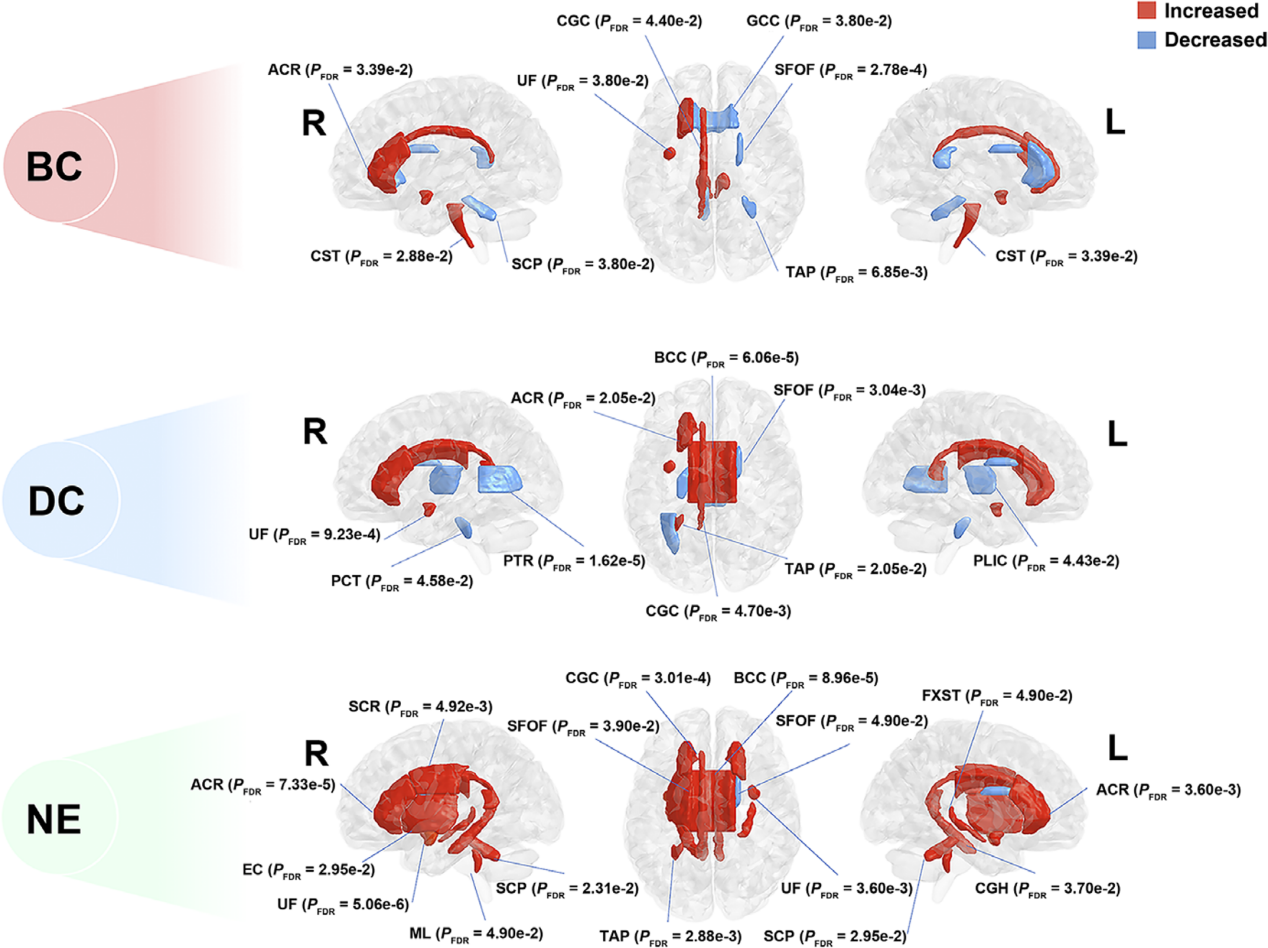
Case–control differences in nodal topological properties of WM-MSNs. Regional brain maps illustrate WM regions showing significant between-group differences in nodal metrics, including BC, DC, and NE. Statistical significance was determined using general linear models adjusted for age, sex, and years of education, with FDR correction (*P* < 0.05). Red and blue colors indicate higher and lower nodal metric values in the major depressive disorder group relative to healthy controls, respectively. Abbreviations for WM regions are provided in Supplementary Table S1. Abbreviations: BC, ‘betweenness centrality’; DC, ‘degree centrality’; FDR, ‘false discovery rate’; L, ‘left hemisphere’; NE, ‘nodal efficiency’; R, ‘right hemisphere’; WM-MSNs, ‘white matter morphometric similarity networks’. Figure 3. long description.

Group-averaged WM-MSNs showed highly comparable overall morphometric similarity between patients with MDD and HCs (MDD: mean ± SD = 0.762 ± 0.110; HC: 0.762 ± 0.116; Figure 2a), indicating largely preserved global similarity patterns across groups. Using a primary threshold of *P* < 0.001 (two-tailed), NBS analysis identified two significant components of altered connectivity (both network-level FWE-corrected *P* < 0.05), comprising 118 connections showing increased morphometric similarity involving 46 WM regions and 45 connections showing decreased morphometric similarity involving 35 WM regions (Figure 2b). At the global level, WM-MSNs in both groups exhibited typical small-world topology (*γ* > 1 and *λ* ≈ 1). Relative to HCs, patients with MDD exhibited higher *γ*, *σ*, Eglob, and Eloc, but lower *λ* (all FDR-adjusted *P* < 0.05; Figure 2c). At the nodal level, widespread topological alterations were observed, with significant between-group differences in BC across 9 regions, DC across 9 regions, and NE across 16 regions (all FDR-adjusted *P* < 0.05; Figure 3).

Associations between WM-MSN alterations and depressive symptom severity were further examined within the MDD group. No edge-level or global network metrics showed significant correlations with HAMD-17 scores after correction for multiple comparisons. In contrast, two nodal properties demonstrated significant symptom-related associations. Specifically, BC of the right superior cerebellar peduncle and NE of the left uncinate fasciculus and right tapetum were significantly associated with greater depression severity (all FDR-adjusted *P* < 0.05). These findings indicate that depressive symptom burden in MDD is primarily related to localized disruptions in regional WM network topology rather than to global network organization or distributed interregional similarity patterns.

### Medication-status subgroup differences

Exploratory analyses were conducted to examine whether medication status was associated with variations in WM-MSN organization. The medicated and unmedicated first-episode MDD subgroups differed significantly in age, years of education, and illness duration, whereas sex distribution was comparable (Supplementary Table S3). NBS analysis revealed limited but detectable connectivity variations between the two subgroups (Supplementary Figure S2). Despite these differences, both groups retained typical small-world network organization. Medicated patients exhibited relatively higher clustering and efficiency-related measures, whereas normalized characteristic path length did not differ significantly (Supplementary Figure S3). Modest regional variations in nodal topology were also observed across several WM tracts (Supplementary Table S4). Given the clinical heterogeneity between subgroups and the exploratory nature of these analyses, these findings should be interpreted cautiously and are presented primarily to contextualize the main case–control results.

### Transcriptional correlates of WM network alterations

**Figure 4.**
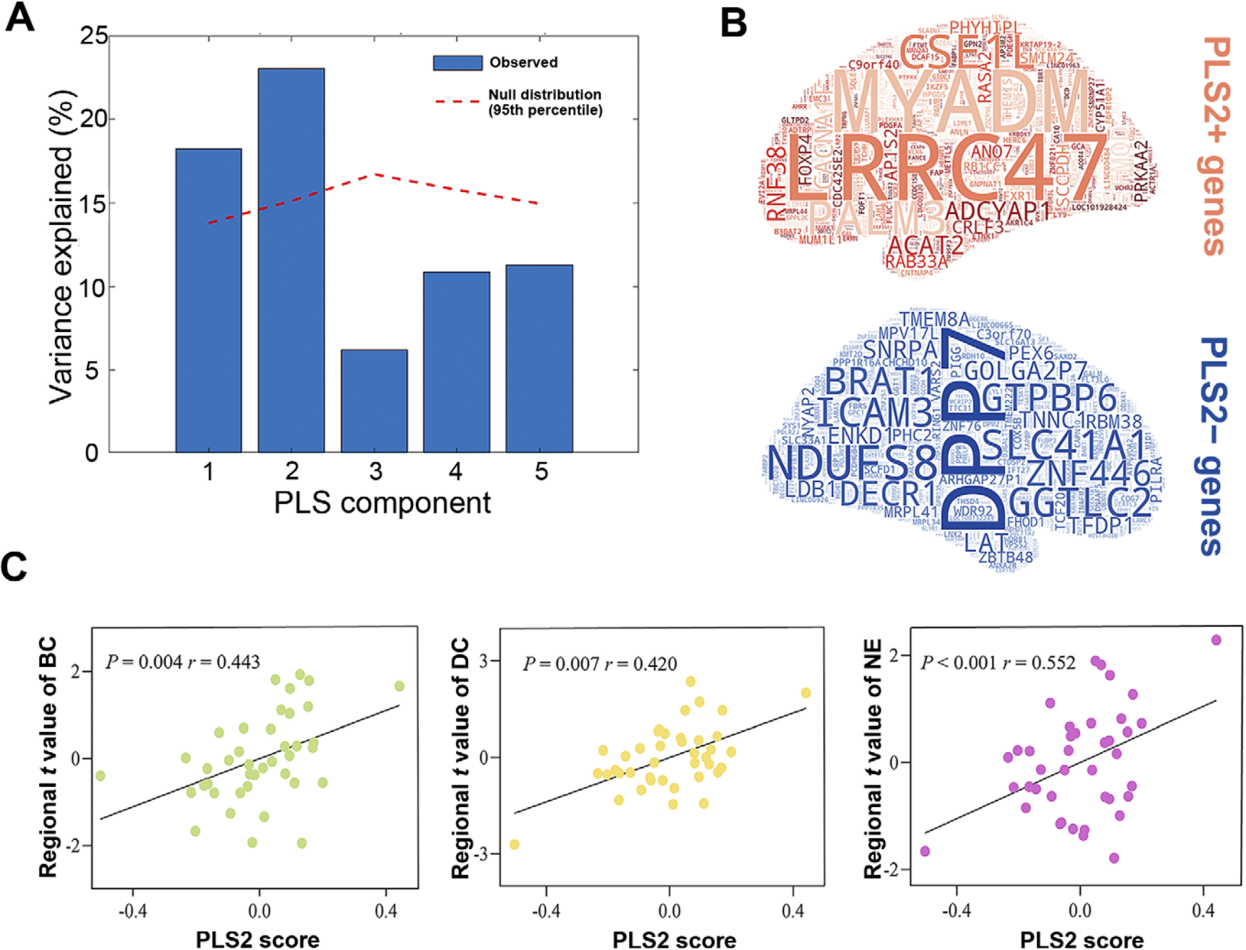
Transcriptomic signatures underlying alterations in WM network topology identified using PLS analysis. (a) Percentage of variance in the nodal alteration patterns explained by each PLS component. The red dashed line indicates the 95th percentile of variance explained under the null distribution derived from spatial permutation testing. PLS2 explained the largest proportion of variance and remained significant following spatial permutation testing using BrainSMASH (1,000 iterations). (b) Word clouds illustrating genes with significant positive (PLS2+, red) and negative (PLS2−, blue) weights on the PLS2 component. Gene name size reflects the magnitude of the corresponding *Z* value. (c) Scatter plots showing significant positive Pearson correlations across WM regions between PLS2 scores and case–control differences in nodal topological metrics, including BC, DC, and NE. Abbreviations: BC, ‘betweenness centrality’; DC, ‘degree centrality’; NE, ‘nodal efficiency’; PLS, ‘partial least squares’; WM, ‘white matter’. Figure 4. long description.

Following preprocessing of the AHBA dataset, regional gene expression profiles were available for 40 WM regions covering 15,633 genes. Spatial permutation testing indicated that two latent components showed significant gene-neuroimaging correspondence. Among these, PLS2 accounted for the greatest proportion of variance in the nodal alteration patterns (23.03%) and was therefore retained for subsequent analyses (Figure 4a). Based on bootstrap-derived gene weights with FDR correction, 2,393 genes were significantly associated with this component, including 672 positively weighted genes (PLS2+) and 1,721 negatively weighted genes (PLS2−) (Figure 4b; Supplementary Table S5). Regional PLS2 scores showed significant positive correlations with nodal alterations in BC (*r* = 0.443, *P* = 0.004), DC (*r* = 0.420, *P* = 0.007), and NE (*r* = 0.552, *P* < 0.001) (Figure 4c), indicating spatial correspondence between transcriptional variation and WM network reorganization in MDD.

**Figure 5.**
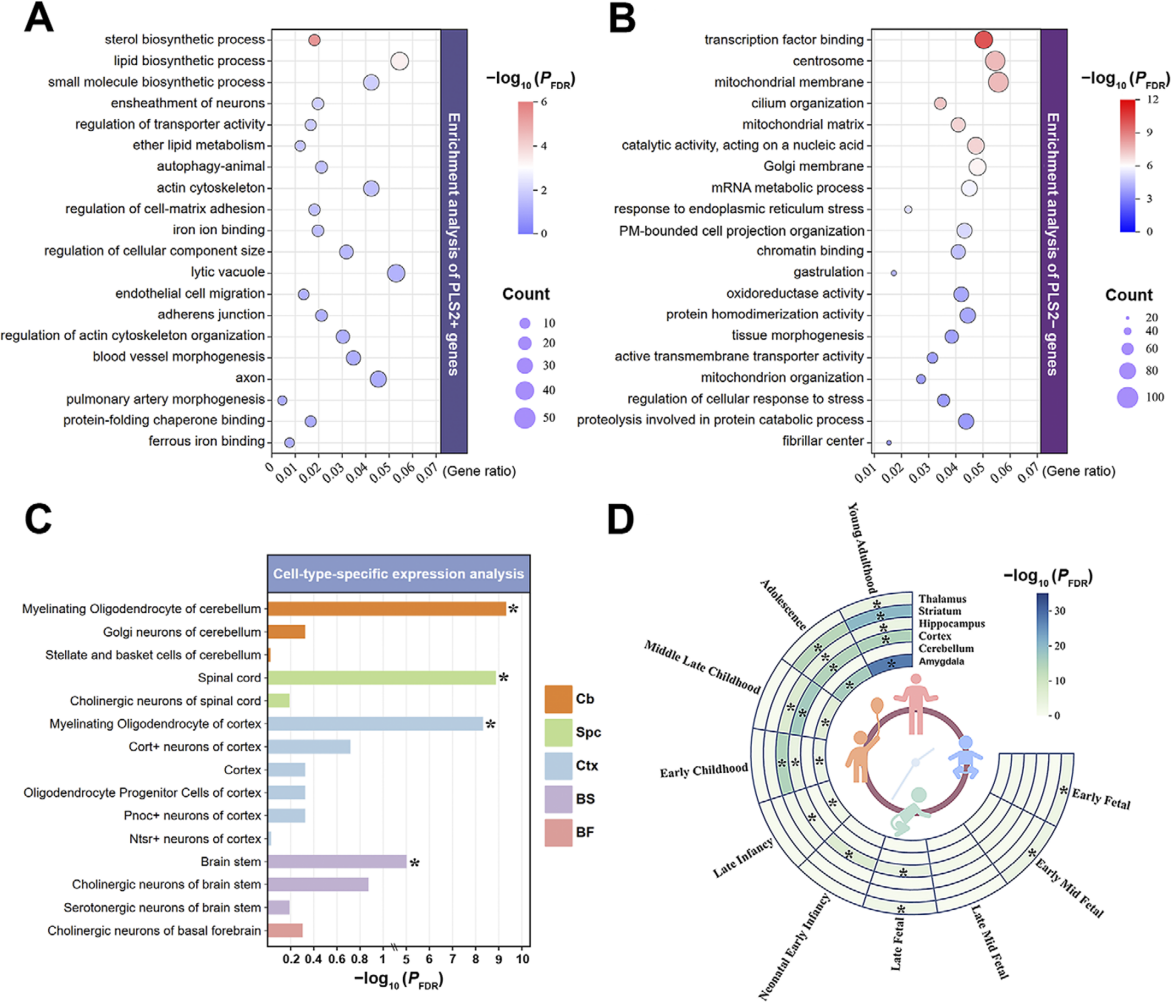
Biological enrichment profiles associated with gene sets weighted by the PLS2 component. (a, b) Functional enrichment analysis of genes with positive (PLS2+) and negative (PLS2−) weights on the PLS2 component. Bubble plots display the top enriched biological terms. The *x*-axis indicates gene ratio, bubble size represents the number of genes contributing to each term, and color denotes −log_10_-transformed FDR-adjusted *P* values. (c) Cell-type-specific transcriptional enrichment across brain regions. The *y*-axis lists distinct neuronal and glial cell populations, and the *x*-axis shows −log_10_-transformed FDR-adjusted *P* values. Asterisks (*) indicate enrichment surviving FDR correction (*P* < 0.05). (d) Spatiotemporal expression patterns of PLS2-weighted genes across developmental stages and brain regions. Concentric rings represent developmental periods ranging from early fetal stages to adulthood, and radial sectors correspond to major brain regions. Color intensity reflects −log_10_-transformed FDR-adjusted *P* values, with asterisks (*) denoting FDR-significant enrichment. Abbreviations: BF, ‘basal forebrain’; BS, ‘brain stem’; Cb, ‘cerebellum’; Ctx, ‘cortex’; FDR, ‘false discovery rate’; KEGG, ‘Kyoto Encyclopedia of Genes and Genomes’; PLS, ‘partial least squares’; PM, ‘plasma membrane’; Spc, ‘spinal cord’. Figure 5. long description.

Functional enrichment analyses of PLS2-associated genes revealed robust overrepresentation across multiple GO categories and KEGG pathways. Positively weighted genes (PLS2+) were significantly enriched in biological processes related to lipid and sterol biosynthesis, neuronal ensheathment, axonal development, cytoskeletal organization, and cell adhesion, as well as molecular functions involving iron ion binding and cellular structural regulation (Figure 5a; Supplementary Table S6). KEGG pathway analysis further indicated enrichment in signaling and metabolic pathways associated with cellular growth, vascular morphogenesis, and autophagy-related processes. In contrast, negatively weighted genes (PLS2−) showed significant enrichment in pathways associated with mitochondrial organization, Golgi-related transport, transcriptional and chromatin regulation, endoplasmic reticulum stress responses, oxidative metabolism, and protein processing and degradation (Figure 5b; Supplementary Table S6). Cell-type-specific expression analysis demonstrated that PLS2+ genes were significantly overrepresented in oligodendrocyte-related cell populations, including CNP-expressing cells in both cortical and cerebellar tissues, suggesting preferential association with myelin-related cellular processes (Figure 5c). No significant enrichment for specific neuronal or glial subtypes was observed for PLS2− genes after FDR correction. Spatiotemporal expression analysis further revealed that PLS2+ genes exhibited broad enrichment across developmental stages ranging from prenatal periods to adulthood, with spatial preference in cortical, limbic, and subcortical regions, including the amygdala, hippocampus, striatum, and thalamus (Figure 5d). In contrast, PLS2− genes did not demonstrate clear developmental stage specificity or regional enrichment patterns.

In addition to the dominant PLS2 component, we further examined transcriptional patterns associated with PLS1, which also showed significant spatial correspondence under permutation testing. Genes with significant weights on PLS1 are provided in Supplementary Table S5, and their functional enrichment results are summarized in Supplementary Table S7.

### Validation analyses

Networks reconstructed from spatially smoothed WMV maps showed strong spatial correspondence with those derived from unsmoothed data in both groups (all *P* < 0.001; Supplementary Figure S4). Edge-level analyses based on smoothed data identified connectivity alterations largely consistent with the primary results, with substantial overlap in altered connections and highly similar regional involvement (Supplementary Figure S5). At the global and nodal levels, however, only partial consistency with the main results was observed: at the global level, a nominal between-group difference was detected only in Eglob (uncorrected *P* = 0.048), while at the nodal level, 8 ROIs showed altered DC and 7 ROIs showed altered NE (Supplementary Figure S6). Repeating all analyses without covariate adjustment yielded highly comparable findings, with extensive overlap in edge-level alterations (Supplementary Figure S7), preservation of the global topological profile, and unchanged spatial patterns of nodal abnormalities (Supplementary Tables S8 and S9).

## Discussion

To our knowledge, this is the first large-scale study to characterize WM-MSNs in MDD at the individual level and to integrate these network alterations with spatial transcriptomic architecture. Our findings indicate that MDD is associated with distributed reconfiguration of macroscale WM network topology, while overall morphometric similarity remains largely preserved. Importantly, the observed network alterations were systematically aligned with regional gene expression gradients related to oligodendrocyte function, myelination, lipid and sterol metabolism, axonal organization, cellular stress responses, and metabolic regulation, with implicated genes also showing broad developmental-stage enrichment. These results suggest that WM abnormalities in MDD reflect coordinated systems-level changes shaped by underlying molecular architecture, thereby extending current neurobiological models by situating WM network disruption within a cross-scale framework that links macroscale organization and transcriptional patterning.

Consistent with a network-level model of depression, the present findings demonstrate that WM-MSNs in MDD are characterized by distributed alterations encompassing both increased and decreased interregional similarity. The coexistence of hyper- and hypo-connected subnetworks suggests that WM network reorganization in MDD does not reflect uniform structural degradation but rather a complex redistribution of morphometric coordination across large-scale circuits. At the global level, the observed pattern of altered small-world organization – including increased clustering and efficiency together with reduced normalized path length – may indicate a shift toward a relatively more integrated and less segregated topological configuration, rather than a simple enhancement of canonical small-world balance. Such alterations may reflect redistribution of morphometric coordination across WM systems supporting large-scale communication, potentially influencing the balance between network segregation and integration (Li et al., 2020; Seguin, Sporns, & Zalesky, 2023). Unlike diffusion-based metrics that primarily capture microstructural properties of individual tracts, WM-MSNs provide a macroscale representation of coordinated morphometric architecture, suggesting that depression-related WM abnormalities extend beyond tract-specific disruptions to involve system-level reconfiguration of structural communication pathways (Cui et al., 2024). These network-level changes may therefore represent emergent properties of distributed WM remodeling that could contribute to altered information transfer efficiency and dysregulated large-scale neural coordination in MDD (Cai et al., 2025; Pei et al., 2025). From a translational perspective, such macroscale organizational features may offer potential structural priors for future data-driven or machine learning frameworks aimed at individualized characterization and risk stratification in depression (Khajehnejad et al., 2025; Mencattini et al., 2025).

At the nodal level, widespread alterations in BC, DC, and NE indicate that multiple WM regions exhibit shifts in their topological importance within WM-MSNs, reflecting reconfiguration of regional contributions to large-scale network organization. Notably, the most prominent alterations were observed in major commissural and association pathways, including the corpus callosum, tapetum, cingulum, and uncinate fasciculus, suggesting that depression-related WM changes preferentially affect circuits supporting interhemispheric coordination and fronto-limbic integration. Rather than reflecting isolated tract-specific abnormalities, these nodal disruptions point to altered roles of key relay regions that facilitate communication across distributed neural systems (Sobral et al., 2025). From a network perspective, such changes may signal reduced efficiency in coordinating information flow between cognitive control and affective processing networks, consistent with models proposing dysregulated large-scale integration in MDD (Jiang et al., 2019). Importantly, the distributed pattern of altered nodal centrality and efficiency supports the view that WM pathology in depression involves coordinated reorganization of structural communication hubs, potentially contributing to impaired synchronization across interacting neural systems involved in emotion regulation, memory, and executive function (Alagapan et al., 2023; Coenen et al., 2019; Lyden et al., 2014; Yu, Sun, & Xia, 2025). These altered nodal regions may also provide a structural basis for identifying candidate targets in future brain–computer interface and neuromodulation frameworks aimed at monitoring or restoring dysfunctional large-scale communication in MDD (Jia et al., 2024; Lan et al., 2025).

Associations between WM-MSN alterations and depressive symptom severity were modest and primarily confined to nodal-level measures, with no significant relationships observed at the edge or global network levels after correction for multiple comparisons. This pattern suggests that variation in current clinical symptom burden may be more closely linked to regionally specific shifts in network centrality than to large-scale changes in overall WM network organization. The limited magnitude of these correlations is consistent with the notion that macroscale WM topology may index relatively stable neurobiological characteristics, whereas HAMD-17 scores reflect fluctuating symptom states captured at a single assessment (Seitzman et al., 2019). Moreover, the multidimensional and heterogeneous clinical presentation of MDD may reduce the sensitivity of composite severity scales to detect direct relationships with distributed network features (Tang et al., 2025). Accordingly, these findings should be interpreted as indicating localized and state-related associations rather than global coupling between WM-MSN organization and overall symptom severity. Although exploratory medication-status analyses suggested additional modulation of WM-MSN topology, these findings should be interpreted cautiously because of potential confounding clinical differences and were therefore not the primary focus of the present study.

Given the substantial polygenic architecture of MDD and its moderate heritability (Adams et al., 2025; Liu et al., 2024; Zhao et al., 2025), integrating spatial transcriptomic information provides a valuable framework for linking distributed WM network alterations to underlying molecular processes. In the present study, regional gene expression patterns showed systematic spatial correspondence with nodal disruptions in WM-MSNs, suggesting that macroscale reorganization of WM topology is constrained by underlying transcriptional gradients. Notably, PLS2+ genes were enriched in lipid metabolism, myelination, axonal ensheathment, and cytoskeletal organization, indicating that coordinated regulation of membrane composition and oligodendrocyte-related processes may contribute to structural remodeling of WM networks in depression. These pathways are closely related to the maintenance of myelin integrity and axonal stability, both of which are essential for efficient large-scale structural communication (Goes et al., 2025; Mocking et al., 2021). Conversely, PLS2− genes were enriched in processes related to mitochondrial function, cellular stress responses, and transcriptional regulation, pointing to potential vulnerability of WM systems to metabolic dysregulation and impaired cellular homeostasis. Rather than representing isolated mechanisms, these molecular processes may jointly influence the balance between structural maintenance and adaptive network reorganization, thereby contributing to altered patterns of network integration observed in MDD (Geng et al., 2024; Scaini et al., 2022; Sforzini et al., 2025; Van Der Spek et al., 2023). In contrast, although PLS1 explained less variance in the nodal alteration patterns than PLS2, it captured additional transcriptional signal and is therefore interpreted as complementary, potentially reflecting broader neuronal signaling and developmental regulatory influences that may indirectly relate to WM network organization (Fries, Saldana, Finnstein, & Rein, 2023).

Additional enrichment analyses provided further context for the molecular processes associated with WM-MSN alterations. Cell-type-specific expression analysis indicated that PLS2+ genes were preferentially enriched in oligodendrocyte-related populations, including CNP-expressing cells across cortical and cerebellar tissues, highlighting the potential relevance of myelin-associated biological pathways to macroscale WM network organization. These findings are broadly consistent with evidence implicating oligodendrocyte dysfunction and altered myelin maintenance in the neurobiology of depression, suggesting that coordinated disruptions in glial support processes may contribute to large-scale structural communication changes (Clayton & Tesar, 2021; Tang et al., 2021). Spatiotemporal expression analyses further showed that PLS2+ genes exhibited relatively stable enrichment across developmental stages from prenatal periods to adulthood, with prominent expression in cortical, limbic, and subcortical regions. Rather than implying a specific developmental insult, this pattern may indicate that transcriptional programs relevant to myelin regulation and network maturation remain influential across the lifespan, potentially shaping vulnerability of fronto-limbic and cortico-striatal-thalamic systems implicated in affective and cognitive regulation (Huang et al., 2022; Kang et al., 2011; Kebets et al., 2021). In contrast, PLS2− genes showed no clear enrichment for specific cell types or developmental windows, supporting the interpretation that these genes may reflect more generalized molecular processes related to cellular homeostasis rather than regionally specialized neurodevelopmental mechanisms.

Validation analyses demonstrated that the principal WM-MSN findings showed different degrees of robustness across validation strategies. Spatial correspondence between smoothed and unsmoothed networks, together with largely overlapping NBS components, suggested that the main edge-level case–control differences were not solely driven by spatial smoothing. However, analyses based on smoothed data showed only partial consistency with the primary results at the global and nodal levels. Some findings, including nodal alterations in several major commissural and association tracts such as the corpus callosum, cingulum, tapetum, and uncinate fasciculus, remained detectable following spatial smoothing. These results suggest that topological properties of WM-MSNs may be sensitive to preprocessing strategies, particularly spatial smoothing, underscoring the need for caution when selecting preprocessing procedures during network construction (Wang, Jin, Zhang, & Wang, 2016). In contrast, analyses repeated without covariate adjustment yielded highly comparable findings, supporting the robustness of the principal results to variations in model specification. Collectively, these findings support the reliability of the present results and suggest that individualized WM-MSN metrics may serve as reproducible markers of large-scale structural network architecture in MDD.

Several limitations should be considered when interpreting the present findings. First, regional gene expression profiles were derived from the AHBA database, which is based on a limited number of adult donors and may not fully capture population-level variability or developmental heterogeneity in transcriptional architecture. Second, although spatially constrained permutation testing was employed to mitigate the influence of spatial autocorrelation, the observed imaging-transcriptomic associations remain correlational and should not be interpreted as evidence of causal molecular mechanisms underlying WM network alterations. Third, the clinical heterogeneity of MDD was not explicitly modeled through stratification by illness stage, course, or comorbidity, which may have influenced the magnitude and spatial distribution of network effects. Fourth, the present study focused primarily on neurobiological and transcriptional correlates of WM network organization and did not incorporate environmental exposures or gene–environment interactions, which are known to play important roles in depression vulnerability and may contribute to individual differences in WM network architecture (Liu et al., 2023; Zhang et al., 2025). Finally, the cross-sectional design limits inferences regarding the temporal evolution of WM network reorganization, and future longitudinal and multimodal investigations will be important for clarifying how structural network alterations relate to disease progression and functional outcomes.

## Conclusions

The present findings highlight that MDD is characterized by a coordinated reorganization of macroscale WM network architecture at the individual level. Altered network topology and region-specific connectivity patterns in major integrative pathways were consistently evident. Importantly, the spatial alignment between WM network alterations and transcriptional gradients related to myelin regulation, metabolic balance, and cellular resilience suggests that depression-related structural network changes may emerge from multilevel biological constraints spanning molecular and systems scales. By situating WM network abnormalities within a cross-scale neurobiological framework, this study contributes to a more integrative understanding of how distributed structural organization relates to the complex pathophysiology of MDD.

## Supplementary Material

The supplementary material for this article can be found at https://zenodo.org/records/17897250.

## Figure 1. Long description

Panel A at the top left shows red and blue icons representing M D D and healthy controls, with arrows leading to M R I scanners labeled 23 sites. Next, brain slices are shown with colored overlays, labeled as parcellating W M V into J H U dash 48. An arrow points to a 48 by 48 W M dash M S N matrix. Further arrows lead to two brain network diagrams labeled global and nodal topological organization, and a violin plot comparing case-control differences with a P value of 0.0032. Panel B at the middle left shows a scatterplot with H A M D dash 17 scores on the x axis and topological organization on the y axis, labeled clinical correlation. Panel C at the middle right shows a schematic brain with red dots, labeled A H B A dataset, an adjacent heatmap labeled gene expression matrix, and a brain map labeled mapping to J H U dash 48. Panel D at the bottom shows a D N A helix and brain labeled gene-imaging association, with an arrow to illustrations of cell types and icons for G O and K E G G pathways, labeled enrichment analyses. All steps are connected by arrows indicating workflow from neuroimaging to gene enrichment.

Navigate back to Figure 1..

## Figure 2. Long description

Panel A at the top left shows two square heatmaps labeled M D D and H C s, each with axes numbered 10 to 40 and a color bar below ranging from 0.4 in blue to 1 in red, representing morphometric similarity between white matter regions. The diagonal is darkest, indicating highest self-similarity, with off-diagonal blocks showing regional patterns. Panel B at the top right is a circular network diagram with regions of interest labeled around the perimeter, colored gray for bilateral, cyan for left, and purple for right hemisphere. Orange lines inside the circle indicate increased similarity edges in M D D, while green lines indicate decreased similarity. The thickness of each segment reflects the number of altered edges per region. Panel C at the bottom contains five side-by-side violin plots comparing M D D (purple) and H C (orange) for the area under the curve of gamma, lambda, sigma, E glob, and E loc. Each plot displays a P sub F D R value above, with M D D generally showing lower or higher distributions depending on the metric. The y-axes are labeled with metric-specific ranges, and the x-axes list the metric names.

Navigate back to Figure 2..

## Figure 3. Long description

From top to bottom, the panels represent B C (betweenness centrality), D C (degree centrality), and N E (nodal efficiency). Each panel contains three brain views: right sagittal, coronal, and left sagittal. Red overlays mark regions with increased nodal metric values in the major depressive disorder group, while blue overlays indicate decreased values. In the B C panel, regions such as A C R (P sub F D R = 3.39 e minus 2), C S T (P sub F D R = 2.88 e minus 2), C G C (P sub F D R = 4.40 e minus 2), G C C (P sub F D R = 3.80 e minus 2), U F (P sub F D R = 3.80 e minus 2), S F O F (P sub F D R = 2.78 e minus 4), S C P (P sub F D R = 3.80 e minus 2), and T A P (P sub F D R = 6.85 e minus 3) are labeled with arrows. In the D C panel, highlighted regions include U F (P sub F D R = 9.23 e minus 4), P C T (P sub F D R = 4.58 e minus 2), P T R (P sub F D R = 1.62 e minus 5), B C C (P sub F D R = 6.06 e minus 5), S F O F (P sub F D R = 3.04 e minus 3), T A P (P sub F D R = 2.05 e minus 2), P L I C (P sub F D R = 4.43 e minus 2), and C G C (P sub F D R = 4.70 e minus 3). In the N E panel, regions such as S C R (P sub F D R = 4.92 e minus 3), A C R (P sub F D R = 7.33 e minus 5), E C (P sub F D R = 2.95 e minus 2), U F (P sub F D R = 5.06 e minus 6), M L (P sub F D R = 4.90 e minus 2), C G C (P sub F D R = 3.01 e minus 4), S F O F (P sub F D R = 3.90 e minus 2), S C P (P sub F D R = 2.88 e minus 3), B C C (P sub F D R = 8.96 e minus 5), F X S T (P sub F D R = 4.90 e minus 2), C G H (P sub F D R = 3.70 e minus 2) are indicated. The overlays and labels are distributed across both hemispheres, with statistical significance denoted by P sub F D R values. The color legend in the upper right specifies red as increased and blue as decreased nodal metric values.

Navigate back to Figure 3..

## Figure 4. Long description

The layout contains three main panels. Top left, a vertical bar graph labeled A shows variance explained in percent on the y axis and P L S component numbers one to five on the x axis. Blue bars represent observed variance, with the second bar (P L S 2) reaching the highest value, above a red dashed line indicating the ninety-fifth percentile of the null distribution. Top right, panel B displays two brain-shaped word clouds: the upper cloud in red labeled P L S 2 plus genes, with large gene names such as L R R C 4 7 and M Y A D M, and the lower cloud in blue labeled P L S 2 minus genes, with prominent names like N D U F S 8 and B R A T 1. Bottom row, panel C contains three scatter plots from left to right, each plotting P L S 2 score on the x axis against regional t value on the y axis. The first plot shows B C (betweenness centrality) with a positive trend, P equals 0.004, r equals 0.443. The second plot shows D C (degree centrality), P equals 0.007, r equals 0.420. The third plot shows N E (nodal efficiency), P less than 0.001, r equals 0.552. All scatter plots display positive correlations with fitted trend lines.

Navigate back to Figure 4..

## Figure 5. Long description

Panel A, top left, is a bubble plot with gene ratio on the x-axis and biological processes on the y-axis, showing enrichment for P L S 2 positive genes. Bubble size indicates gene count, color shows negative log ten F D R. Top terms include sterol and lipid biosynthetic processes. Panel B, top right, is a bubble plot with gene ratio on the x-axis and biological terms on the y-axis for P L S 2 negative genes. Top terms include transcription factor binding and centrosome. Panel C, bottom left, is a horizontal bar graph with negative log ten F D R on the x-axis and cell types on the y-axis, colored by brain region: Cb (cerebellum), Spc (spinal cord), Ctx (cortex), BS (brain stem), BF (basal forebrain). Asterisks mark significant enrichment, with myelinating oligodendrocytes of cerebellum and spinal cord showing highest values. Panel D, bottom right, is a radial diagram with concentric rings representing developmental stages from early fetal at the center to adulthood at the perimeter. Radial sectors represent brain regions: thalamus, striatum, hippocampus, cortex, cerebellum, amygdala. Color intensity reflects negative log ten F D R, with asterisks marking significant enrichment in specific regions and periods.

Navigate back to Figure 5..
